# Supplementary material for: Level of shared decision making and associated factors among patients with mental illness in Northwest Ethiopia: Explanatory sequential mixed method study
Source: PLoS One. 2023 Apr 7;18(4):e0283994. doi: 10.1371/journal.pone.0283994 (PMC10081773; doi:10.1371/journal.pone.0283994)
Supplement: S1 Checklist — (DOCX) [file pone.0283994.s001.docx]

S1. STROBE Statement—checklist of items that should be included in reports of observational studies

|  | Item No. | Recommendation | Page  No. | | | | Relevant text from manuscript | | |
| --- | --- | --- | --- | --- | --- | --- | --- | --- | --- |
| **Title and abstract** | 1 | (*a*) Indicate the study’s design with a commonly used term in the title or the abstract | i | | | | Explanatory sequential mixed method study | | |
|  |  | (*b*) Provide in the abstract an informative and balanced summary of what was done and what was found | i i | | | | Shared decision-making is patient–centered and a recovery-oriented mental health system in which consumers are encouraged to actively engage in illness management. Although shared decision-making research in mental health has evolved rapidly in the past two decades, there is a lack of studies examining the level and factors associated with shared decision-making practice in low-resource countries like Ethiopia. | | |
| Introduction | | | | | | |  | | |
| Background/rationale | 2 | Explain the scientific background and rationale for the investigation being reported | | 1 | | Evidence showed that shared decision-making can be successfully practiced in psychiatry would contribute to an improved inclusion of psychiatric patients in therapeutic decisions and thereby help to implement the basic rights of a group of patients who have not sufficiently benefited from consumer empowerment in other medical fields. Non-psychiatric patients were 18 times more likely to prefer options for their treatment and 2 times more likely to prefer to take medical decisions on their own than psychiatric patients.  The primary outcomes of SDM in mental health often differ from outcomes in non-mental health studies (such as SDM use, knowledge, and conflict level), and involve a focus on goals important to the field, such as empowerment, self-determination, and recovery. In mental health care, SDM has been recommended for people with serious mental illness, given that self-determination, choice, and autonomy are core aspects of recovery-oriented care.  Assessment of shared decision-making the outpatient psychiatry department has great importance for improving and changing quality health service delivery by focusing on identifying factors that affect shared decision-making with mental health services.  It will help to increase the treatment-seeking behavior of the patient. In addition, there was no similar research completed in Ethiopia. It helps as a source of data and information for stakeholders and researchers who work in the area of mental health services and those who will conduct further studies on the issue under inquiry | | | |
| Objectives | 3 | State specific objectives, including any prespecified hypotheses | |  | | - To determine level of shared decision-making and associated factors among patients’ with mental illness at Tibebe Ghion specialized and Felege Hiwot comprehensive specialized hospital, North West, Ethiopia, 2022. - To identify factors associated with shared decision-making among patients’ with mental illness at Tibebe Ghion specialized and Felege Hiwot comprehensive specialized hospital, North West, Ethiopia, 2022. - To explore barriers of shared decision-making among patients’ with mental illness at Tibebe Ghion specialized and Felege Hiwot comprehensive specialized hospital, North West, Ethiopia, 2022. | | | |
| Methods | | | | | | | | |  |
| Study design | 4 | Present key elements of study design early in the paper | | | 2 | | | An institutional-based explanatory sequential mixed study design was used | |
| Setting | 5 | Describe the setting, locations, and relevant dates, including periods of recruitment, exposure, follow-up, and data collection | | | 2 | | | This study was conducted at Tibebe Ghion specialized hospital (TGSH) and Felege Hiwot Comprehensive Specialized Hospital (FHCSH) in Bahir Dar city, Ethiopia. TGSH hospital gives mental health services with four outpatient departments, one emergency room, and two outpatients with a total bed of 13. It also gives psychotherapy services by a clinical counselor. The community is served by two psychiatrists, seven mental health specialists, one clinical counselor, and six psychiatric professionals. The estimated number of annual outpatient clients was 4864 (405 per month based on the monthly report of the psychiatry unit). FHCSH also provides mental health services with inpatients (17 beds) and four outpatient departments. It serves a total of 19,200 clients annually (1600 per month). The patient was served by four mental health specialists and six psychiatry professionals. Generally, the monthly patient flow for both hospitals in psychiatry outpatient was 2005 patients. The study was conducted from July 18/2022 to September 18, 2022. | |
| Participants | 6 | (*a*) *Cohort study*—Give the eligibility criteria, and the sources and methods of selection of participants. Describe methods of follow-up  *Case-control study*—Give the eligibility criteria, and the sources and methods of case ascertainment and control selection. Give the rationale for the choice of cases and controls  *Cross-sectional study*—Give the eligibility criteria, and the sources and methods of selection of participants | | | 2 | | | All patients who visited the outpatient department (OPD) for mental health services at TGSH and FHCSH and who were available at the time of data collection and whose age was 18 years or older were included. Those patients who were seriously ill and had no insight were excluded. | |
|  |  | (*b*) *Cohort study*—For matched studies, give matching criteria and number of exposed and unexposed  *Case-control study*—For matched studies, give matching criteria and the number of controls per case | | |  | | |  | |
| Variables | 7 | Clearly define all outcomes, exposures, predictors, potential confounders, and effect modifiers. Give diagnostic criteria, if applicable | | |  | | | Shared Decision-Making - This measured by core instrument consists of nine items, which can be rated on a six-point scale from ‘‘completely disagree’’ (0) to ‘‘completely agree’’ (5). Summing up all items leads to a raw total score between 0 and 45, then transformed to 0 to 100 by multiplying 20/9. The mean was computed and used as a cut point: agree > 54.48 and disagree <54.48.  Perceived compassionate care: - It is essential to high quality health care and it was measured by Schwartz Center Compassionate Care Scale® (SCCCS) which measures level of perceived compassionate health care and it classified as:   - Good: - A score greater than the mean (76.74) considered good compassionate care - Poor: - Score less than the mean (76.74) considered poor compassionate care.   Social Support: - measured by OSLO 3 social support which is categorized into: -   - Poor social support - 3–8 - Moderate social support - 9–11 - Strong social support- 12–14 ([47](#_ENREF_47)).   Perceived stigma: - Measured by Stigma Scale for Receiving Psychological Help, a five-item scale with internal (Cronbach’s alpha of .74) was created by summing the score (0–3) for each item, ranging from 0, lowest perceived stigma, to 15, highest perceived stigma; then the cut point is 6.86. The score above 6.86 were considered as having high perceived stigma and the score below 6.68 considered as having low perceived stigma.  Patient anticipated stigma from health workers: - measured by patient anticipated stigma to health care workers, a four item with a Likert scale ranging from 1 (very unlikely) to 5 (very likely). Summing up all items leads to a total score from 4 to 20. The mean score above 10.89 were considered as high patient anticipated stigma to health workers and the score below 10.89 were considered as low anticipated stigma to health workers. | |
| Data sources/ measurement | 8* | For each variable of interest, give sources of data and details of methods of assessment (measurement). Describe comparability of assessment methods if there is more than one group | | |  | | | 1. Level of perceived compassionate care was measured by a validated 12-item Schwartz Center Compassionate Care Scale® (SCCCS). Each item is scored on a 10-point scale from 1–10 with response options that range from 1 (not at all successful) to 10 (very successful). A total compassion score was calculated, and a total mean was computed (76.74) and considered a cutoff point. A score greater than 76.74 were considered good compassionate care, and less than 76.74 was considered poor compassionate care. Permission to use the scale was granted by Beth A. Lown, the copyright holder of the instrument and the English and Amharic version obtained from Merkeb Zeray, validator of the tool in Ethiopia. The tool was validated in Ethiopia and showed internal consistency (a=0.95) with item-to-total correlations were excellent, ranging from 0.83 to 0.93 and a test- retest reliability of 0.90.. 2. Oslo Social Support Scale (OSSS)—a three-item scale with Cronbach’s alpha of 0.75 and has a range value of 3-14, further categorized as follows: “poor support,” 3–8; “moderate support,” 9–11; and “strong support,” 12–14 with the internal consistency could be regarded as acceptable with α = .640. 3. The 9-item Shared Decision Making Questionnaire (SDM-Q-9)—it is a tool that is used to assess the decision making style of a physician on patient treatment with internal consistency yielded a Cronbach’s (α) of 0.938 ([48](#_ENREF_48)). 4. Perceived stigma:- Measured by Stigma Scale for Receiving Psychological Help, a five-item scale with internal consistency (Cronbach’s alpha of .74) was created by summing the score (0–3)(0 – strongly disagree to 3- strongly agree) for each item, ranging from 0, lowest perceived stigma, to 15, highest perceived stigma. 5. Patient anticipated stigma from health workers: - measured by patient anticipated stigma to health care workers scale, a four item with a Likert scale ranging from 1 (very unlikely) to 5 (very likely) (Cronbach’s α = 0:83). | |
| Bias | 9 | Describe any efforts to address potential sources of bias | | |  | | | The English and Amharic version of the dependent variable questionnaire was obtained from the validator of the tool; validated tool was used. The semi-structured interviewer administered questionnaire for independent variables was prepared in English first and then changed to local language (Amharic) for better understanding of the data collectors and the respondents and then it was changed to English language again to check its consistency. One-day training was given for data collectors on the objective of the study, data collection tools and procedures, how to approach potential respondents and how to keep confidentiality. Before conducting the main study, pretesting on 5% of the sample size (on 21 participants) was done prior to a week of the actual data collection period at Addis Alem General Hospital. Final data collection tool was refined based on the findings from the pretest. The collected data was carefully checked for completeness and consistencies on daily basis. | |
| Study size | 10 | Explain how the study size was arrived at | | | 3 | | | Since no studies have been done in Ethiopia on shared decision-making, we have taken a proportion of 50%. Then, by using a single population proportion formula with a 95% confidence interval and a 5% margin of error.  $n=\frac{(Za/2)2 P\left( 1-P \right)}{d2}$  Z = 95% confidence interval (1.96)  d= 0.05 (margin of error)  $n=\frac{{(1.96)}^{2} 0.5\left( 1-0.5 \right)}{{(0.05)}^{2}}$  $n=384$  After adding a 10% non-response rate, the final sample size was 423.  To get representative data in both hospitals, proportional allocation of the sample was calculated based on patient flow per month as 405/1600, and then the proportion would be 1:4. The final sample size distribution was (423*1/4) 106 for TGSH and the rest (423*3/4) 317 for FHCSH.  For qualitative research, 10 participants for in-depth interviews were selected purposively after analysis of the quantitative data, and then until information saturation was obtained. | |

Continued on next page

| Quantitative variables | 11 | Explain how quantitative variables were handled in the analyses. If applicable, describe which groupings were chosen and why | 5 | Data were collected by Epicollect5 using smart phone and then was directly exported to Statistical Package for social science (SPSS) version 25 for further analysis. |
| --- | --- | --- | --- | --- |
| Statistical methods | 12 | (*a*) Describe all statistical methods, including those used to control for confounding |  |  |
|  |  | (*b*) Describe any methods used to examine subgroups and interactions |  |  |
|  |  | (*c*) Explain how missing data were addressed | 5 | Epicollect5 apps was used. |
|  |  | (*d*) *Cohort study*—If applicable, explain how loss to follow-up was addressed  *Case-control study*—If applicable, explain how matching of cases and controls was addressed  *Cross-sectional study*—If applicable, describe analytical methods taking account of sampling strategy | 5 | Descriptive statistics such as mean, standard deviation, proportions, frequency, and percentage were used to describe the outcome and independent variables. Logistic regression model (both bi-and multi-variable logistic regression) was used to examine factors associated with perceived compassionate care among the study participants. Variables which were show association with dependent variable in the bivariate analyses at P<0.25 were candidate for multivariable logistic regression model. Adjusted odds ratio (AOR) with 95% CI and p-value less than 0.05 was used to declare the strengths and the factors significantly associated with outcome variable respectively. Model fitness was checked, which was (Hosmer and Lemeshow Test) =0.10, no multicolinearity (Tolerance>0.1 and Variance inflation factor (VIF)<3). |
|  |  | (*e*) Describe any sensitivity analyses |  |  |
| Results | | | | |
| Participants | 13* | (a) Report numbers of individuals at each stage of study—eg numbers potentially eligible, examined for eligibility, confirmed eligible, included in the study, completing follow-up, and analysed | 6 | A total of 419 patients participated in this study, with a response rate of 99%. From two hundred six patients with mental illness, 39.3%, 27.2%, 18.9% and 7.8% had poor decision making involvement during their psychiatric outpatient follow-up among patients diagnosed with Schizophrenia, Major Depressive Disorder (MDD), Bipolar disorder (BPI disorder), and Generalized Anxiety Disorder (GAD) respectively |
|  |  | (b) Give reasons for non-participation at each stage |  | Refusal to participate in the study. |
|  |  | (c) Consider use of a flow diagram |  | No flow diagram. |
| Descriptive data | 14* | (a) Give characteristics of study participants (eg demographic, clinical, social) and information on exposures and potential confounders | 6 | Nearly half of the participants were females (50.8%) and more than one-third (37.2 %) of the participants were aged 26–35 years, with a mean age of 33.02 (±SD of 11.2). The majority of the participants (98.6%) were Amhara in ethnicity, nearly half (50.8%) of the participants lived in urban areas, and 26.0 % were unable to read and write. 70.6% were Orthodox Christians, and one-third (30.1%) were farmers. Moreover, nearly half (47.5%) and more than one quarter (28.2%) of the participants were married and had a monthly income <2000 birr (with SD ± 3529 birrs) respectively (table 1). |
|  |  | (b) Indicate number of participants with missing data for each variable of interest |  | No missing value |
|  |  | (c) *Cohort study*—Summarise follow-up time (eg, average and total amount) |  |  |
| Outcome data | 15* | *Cohort study*—Report numbers of outcome events or summary measures over time |  |  |
|  |  | *Case-control study—*Report numbers in each exposure category, or summary measures of exposure |  |  |
|  |  | *Cross-sectional study—*Report numbers of outcome events or summary measures | *9* | Overall, 206 (49.2%) (95% CI = 45.9%-55.7%) of participant’s had low level of clinical decision-making involvement during treatment at TGSH and FHCSH. |
| Main results | 16 | (*a*) Give unadjusted estimates and, if applicable, confounder-adjusted estimates and their precision (eg, 95% confidence interval). Make clear which confounders were adjusted for and why they were included |  |  |
|  |  | (*b*) Report category boundaries when continuous variables were categorized |  |  |
|  |  | (*c*) If relevant, consider translating estimates of relative risk into absolute risk for a meaningful time period |  |  |

Continued on next page

| Other analyses | 17 | Report other analyses done—eg analyses of subgroups and interactions, and sensitivity analyses | | | |  |  |
| --- | --- | --- | --- | --- | --- | --- | --- |
| Discussion | | | | | | | |
| Key results | 18 | Summarise key results with reference to study objectives | 16 | | This study revealed that the low shared decision-making practice at TGSH and FHCSH was 49.2% (95% CI 45.9%-55.7%). This implies that shared decision-making needs high attention as it is essential for patient-centered care ([19](#_ENREF_19)). The finding of this study also showed that low perceived compassionate care, poor social support and no community-based health insurance were associated with low shared decision-making practice.  The level of shared decision making practice in this study was low (49.2%). This was lower than the study done in Public Hospitals of West Showa Oromia, Ethiopia (64.8%) ([17](#_ENREF_17)) and in Australia with 60% reporting experiencing SDM ([11](#_ENREF_11)). This may be due to despite the flourishing development of SDM interventions ([20](#_ENREF_20)), SDM implementation for patients with serious mental illness has been relatively less successful than for other groups. This disparity has been attributed to a variety of barriers ([21](#_ENREF_21)), including clinicians’ paternalistic approach to the care of patients with serious mental illness and stigma-related beliefs that SDM is inappropriate for such patients. Unlike other chronic non-psychiatric conditions, the characteristics of serious mental illness may pose a major barrier to SDM implementation. For example, psychotic symptoms and cognitive deficits associated with schizophrenia can affect decision-making capacity and serve as barriers to SDM, whereas an elevated blood-glucose level associated with diabetes does not stop patients from being involved in SDM ([14](#_ENREF_14), [21](#_ENREF_21)).  This result was higher than a study finding of patient attitude in SDM in outpatient psychiatry services where 30% of patient did not experience shared decision-making involvement ([11](#_ENREF_11)). The variation may be explained by the settings, the study population, and tool differences used in the studies. This study uses the standardized tool which was the 9-item shared decision-making questionnaires but the previous study uses Mini-Mental State Examination in 109 participants. | | |
| Limitations | 19 | Discuss limitations of the study, taking into account sources of potential bias or imprecision. Discuss both direction and magnitude of any potential bias | 16 | | The study’s findings might be prone to a response bias due to the patients self-report, and they do not provide an objective measure of shared decision-making. The questionnaire has some sensitive issues, which may suffer from social desirability. | | |
| Interpretation | 20 | Give a cautious overall interpretation of results considering objectives, limitations, multiplicity of analyses, results from similar studies, and other relevant evidence | 14 | | This study revealed that the low shared decision-making practice at TGSH and FHCSH was 49.2% (95% CI 45.9%-55.7%). This implies that shared decision-making needs high attention as it is essential for patient-centered care. This low level of shared decision making practice in mental health may be due to non-psychiatric patients were 18 times more likely to prefer options about their treatment and 2 times more likely to prefer to take medical decisions on their own than psychiatric patients. Additionally, despite the flourishing development of SDM interventions, SDM implementation for patients with serious mental illness has been relatively less successful than for other groups. This disparity has been attributed to a variety of barriers, including clinicians’ paternalistic approach to the care of patients with serious mental illness and stigma-related beliefs that SDM is inappropriate for such patients. Unlike other chronic non-psychiatric conditions, the characteristics of serious mental illness may pose a major barrier to SDM implementation. For example, psychotic symptoms and cognitive deficits associated with schizophrenia can affect decision-making capacity and serve as barriers to SDM, whereas an elevated blood-glucose level associated with diabetes does not stop patients from being involved in SDM.  This result was higher than a study finding of patient attitude in SDM in outpatient psychiatry services where 30% of patient did not experience shared decision-making involvement. The variation may be explained by the settings, the study population, and tool differences used in the studies. This study uses the standardized tool which was the 9-item shared decision-making questionnaires but the previous study uses Mini-Mental State Examination in 109 participants.  This study showed that patients with mental illness with the low level of perceived compassionate care were 4.45 times (AOR = 4.45, 95% CI 2.52-7.89) more likely to get low shared decision making than those patients with the high level of perceived compassionate care. This finding was supported by the qualitative study findings. As IDI1 stated "... Compassion means treating patients with respect, and the care provided by hospital psychiatrists and mental health professionals is often rough; not customer-centered. Some professionals are empathetic, and some are not. They were not involving us during the treatment process. The main reason for this problem was the negligence of mental health professionals" (18 year old male). This finding might be psychiatrists, as well as other medical providers, score low on scales of patient involvement in decision-making, perhaps in part because traditional genetic-counselling has been based on autonomous choice models and negative internalized self- stigma which affects perceived compassionate care, a common experience that leads patients to underestimate their own competency for decision-making and autonomy.  This study also showed that patients with poor social support were 1.72 times (AOR =1.72, 95% CI 1.06–2.8) more likely to receive low shared decision-making than those patients with strong social support. This study was supported by the qualitative finding as the participant (IDI2) stated that"…One scenario is that I am going to stop taking this medicine because the price of the medicine is high. I did not come up with enough money; no one supported me. As a result, it creates a barrier between me and the health professional’s relationship and it affects shared decision involvement between me and the mental health professional”. This could be due to strong social support being highly associated with good medication adherence, improved quality of life, increased mental health-seeking behavior, improved treatment outcomes, and increased health professional- to-patient bonding Emotional support includes shared risk-taking and intimacy as well as the provision of caring and empathy. A greater barrier, though, may be clinicians’ fear of liability and legal exposure. Clinicians may resist SDM with patients with serious mental illness because they fear being held liable for any potentially negative outcome that might result from SDM, such as symptom exacerbation, hospitalization, or death. Burnout, patient load, and limited appointment time also contribute to clinicians’ reluctance to engage in SDM. This study also indicates that patients who had no community based health insurance were 1.96 times (AOR =1.96, 95% CI 1.04–63.69) more likely to receive low shared decision-making than those who had community-based health insurance. This finding was explained by the qualitative findings as community-based health insurance helps the patient to choose the preferred treatment without financial constraints. As IDI7 explains,"...because of I have had community-based health insurance, I decided together with the experts to accept the medicine given by the expert, and I feel happy that I decided on every aspect with them (**IDI7)**. This finding may be due to the utilization of health services among insured households with community-based health insurance being higher and is an emerging strategy for providing financial protection against health-related poverty and fostering shared decision making. | | |
| Generalisability | 21 | Discuss the generalisability (external validity) of the study results |  | | This study used the maximum sample size to assure the generalizability of the result. | | |
| Other information | |  | | | | | |
| Funding | 22 | Give the source of funding and the role of the funders for the present study and, if applicable, for the original study on which the present article is based | 17 | No funds to conduct this research. | | | |

*Give information separately for cases and controls in case-control studies and, if applicable, for exposed and unexposed groups in cohort and cross-sectional studies.

**Note:** An Explanation and Elaboration article discusses each checklist item and gives methodological background and published examples of transparent reporting. The STROBE checklist is best used in conjunction with this article (freely available on the Web sites of PLoS Medicine at http://www.plosmedicine.org/, Annals of Internal Medicine at http://www.annals.org/, and Epidemiology at http://www.epidem.com/). Information on the STROBE Initiative is available at www.strobe-statement.org.
