# Supplementary material for: Level of shared decision making and associated factors among patients with mental illness in Northwest Ethiopia: Explanatory sequential mixed method study
Source: PLoS One. 2023 Apr 7;18(4):e0283994. doi: 10.1371/journal.pone.0283994 (PMC10081773; doi:10.1371/journal.pone.0283994)
Supplement: Attachment — Submitted filename: Rebuttal Letter.docx [file pone.0283994.s003.docx]

**Rebuttal letter to academic editor and the reviewers**

**A point-by-point response for academic editor**

**Dear academic editor**

First, we would like to express our deepest gratitude for your constructive and valuable comments and suggestions to the best quality of our manuscript. As per your comments and suggestions, corrections have been made to the manuscript accordingly.

**Comment 1:** “Please ensure that your manuscript meets PLOS ONE's style requirements, including those for file naming. The PLOS ONE style templates can be found athttps://journals.plos.org/plosone/s/file?id=wjVg/PLOSOne_formatting_sample_main_body.pdf and<https://journals.plos.org/plosone/s/file?id=ba62/PLOSOne_formatting_sample_title_authors_affiliations.pdf>”

**Authors’ response:** Thanks for your great comments. We have revised the manuscript as per the PLOS ONE's style requirements.

**Comment 2: “**In your Data Availability statement, you have not specified where the minimal data set underlying the results described in your manuscript can be found. PLOS defines a study's minimal data set as the underlying data used to reach the conclusions drawn in the manuscript and any additional data required to replicate the reported study findings in their entirety. All PLOS journals require that the minimal data set be made fully available. For more information about our data policy, please see http://journals.plos.org/plosone/s/data-availability”.

**Authors’ response:** According to your constructive comments, all data are fully available without restriction.

**Comment 3: “**Your ethics statement should only appear in the Methods section of your manuscript. If your ethics statement is written in any section besides the Methods, please delete it from any other section”.

**Authors’ response:** We appreciate your great concern. Some reptations were omitted.

**Comment 4:** Please include a caption for figure 1.

**Authors’ response:** Thankfully, we have corrected it. Caption is included in figure 1.

**Comment 5: “**Please ensure that you refer to Figure 1 in your text as, if accepted, production will need this reference to link the reader to the figure”.

**Authors’ response:** Thankfully, we have corrected it.

**Comment 6:** “We note you have included a table to which you do not refer in the text of your manuscript. Please ensure that you refer to Table 3 in your text; if accepted, production will need this reference to link the reader to the Table”.

**Authors’ response:** Thankfully, we have corrected it.

**Comment 7:** “Please include captions for your Supporting Information files at the end of your manuscript, and update any in-text citations to match accordingly. Please see our Supporting Information guidelines for more information: http://journals.plos.org/plosone/s/supporting-information”.

**Authors’ response:** Thankfully, we have corrected it. Captions are included for the supporting information file in the revised manuscript.

**A point-by-point response for Reviewer 1**

**Dear Reviewer**

First of all, we would like to express our heartful thanks to the corrections, comments and suggestions you have forwarded for improving the quality of our manuscript. Dear reviewer, we have accordingly incorporated the whole things that you have raised and you can recheck the corrections made in the revised manuscript and the response letter attached.

**Comment 1:** The authors are to be congratulated for taking on this important subject. Shared decision-making between psychiatric patients and mental health workers is as important in Ethiopia as it is in England or America. The major impediment to achieving shared decision-making everywhere is the constraint of time; the less time per patient, the more difficult it is to achieve. Shared decision-making assumes that patients believe that there is something wrong and therefore need for decisions regarding treatment. If patients have an impaired awareness of their illness – anosognosia - shared decision-making is impossible to achieve other than agreeing to do nothing.
The main shortcoming of this is that it does not deal with this issue sufficiently. It simply says that “those patients who were seriously ill and had no insight were excluded.” How many were excluded? What percentage of the total sample?

**Authors’ response**: - By thanking you for your constructive comment, we have corrected it. Four patients were excluded due to they did not give response due to their active psychopathology. They were referred to the Emergency Psychiatry ward.

**b. “**Various levels of impaired insight occur most commonly in psychosis. Therefore if a mental health worker perceives or believes that a given patient has impaired insight, the mental health worker is less likely to attempt to achieve shared decision-making. If this is true that I would expect shared decision-making to be lower for patients with a diagnosis of schizophrenia, for example, and for those with a diagnosis of depression or anxiety disorders, for example. This could be easily tested with your existing data”

**Authors, response:** We really appreciate your suggestions to boost the quality of our manuscript. Based on your suggestion, the manuscript is revised. From two hundred six patients with mental illness, 39.3%, 27.2%, 18.9% and 7.8% had poor decision making involvement during their psychiatric outpatient follow-up among patients diagnosed with Schizophrenia, Major Depressive Disorder (MDD), Bipolar disorder (BPI disorder), and Generalized Anxiety Disorder (GAD) respectively (Fig 2).

**Comment 2: “**The paper could be improved grammatically by using a native English speaker. And there are a few typos such as ages listed in table 1 and the lack of publication information for reference number 16.

**Authors’ response:** We are very much thankful for your comment. We have corrected it accordingly. DOI of Reference number 16 is https://psycnet.apa.org/doi/10.2975/34.1.2010.7.13

**A point-by-point response for Reviewer 2**

**Dear Reviewer**

First of all, we would like to express our heartfelt thanks to the corrections, comments and suggestions you have forwarded for improving the quality of our manuscript. Dear reviewer, we have accordingly incorporated the whole things that you have raised and you can recheck the corrections made in the revised manuscript and the response letter attached.

**Comment 1: “**The introduction is not clear enough. In this sense, you need to rewrite your introduction. I also think that there needs to be more clarity about the justification of the study and why you chose to use explanatory sequential mixed method. In general, the authors should revise their manuscript in a scientific way with correct language usage and resubmit their manuscript. Therefore, you should rewrite your introduction by focus on your topic taking into consideration the following points that to be raised. 1. What is it? The problem, 2.Magnitude and distribution?, 3. Possible factors?, 4. Severity and consequences? and 5. Gap statement?”

**Authors’ response:** We are very much thankful for your comment. We modified it based on your great suggestion.

**Comment 2: “**Keywords: Minor edition; make the first letters of all keywords uppercase.”

**Authors’ response:** We are so thankful for your insight. We have corrected it accordingly.

**Comment 3:** Methods

a. Study design?

**Authors’ response:** We are very much thankful for your comment. We have used Explanatory Sequential Mixed Method study. Since there were limited studies regarding shared decision making in mental health, its aim is to use qualitative approach to explain and support quantitative results.

b. No needs to present sample size formula rather narrate how the sample size calculated. Remove the formula and definition of symbols or alphabet used in the sample size calculation.
   **Authors’ response:** We are very much thankful for your comment; we modified it based on your great suggestion.
c. Figure 1 is the not figure in the scientific writing. Please, recreate figure 1 in attractive way by arranging the alignment of arrow and rectangles used.

**Authors, response:** We really appreciate your suggestions to boost the quality of our manuscript. Based on your suggestion, we have modified it.

d. Where are study variables?
   Operational definition: If you present operational definition for the outcome variable “Shared decision-making (level)” (What it mean low or high Shared decision making) in methodology part is more appropriate for scientific paper, and the way you measured the outcome variable (scoring, including categorization and how composite index or total score formed) under data collection with the variables measured. Thus, I recommend presenting the operational definition of Shared decision making level, and providing the method of measuring the Shared decision-making. Most importantly provide your justification why mean score was used as cut-off point with reference. Who recommend the cut-off point?
   **Authors’ response**: With heartfelt thanks, study variables and operational definitions of each variable are added in the revised manuscript. Since it is a continuous variable the validator of the tool recommends to use the mean score (the reference is cited in the tool of the outcome variable https://doi.org/10.1016/j.pec.2009.09.034)

**Comment 4**: Result

a. Change Quantitative finding to socio-demographic characteristic of the respondents **Authors’ response:**

**Authors’ response:** We are very much thankful for your comment. It was writing error and we have corrected it accordingly.

b. If you are presenting the exact number remove the words “about, nearly, majority…”, Because you are writing the exact number and the reader can evaluate by themselves.

**Authors’ response:** We are very much thankful for your insight. We have corrected it accordingly

c. Results presentation is not presented in the way readers can understand. Just a list of sentences without creativity. The authors should read previously published similar article and represent the results part clearly.

**Authors, response:** We really appreciate your suggestions to boost the quality of our manuscript. Based on your suggestion, we have modified it. The result is presented in tables, sentences and figures.

**Comment 5:** Discussion

The discussion part is not presented in the way guide the readers and clear way. In the first part of the discussion part, the authors should summarize the key findings which they are going to discuss. However, the authors just stated their discussion by comparing their finding with the previous findings. Even the way they are comparing with previous finding is not clear. Thus, the authors should read the previous study or any scientific writing books and try to summarize the key findings in the first paragraph of the discussion and then start results interpretation, and compare and contrast with previous studies. I strongly recommend for the authors should follow the previous study and try to present the discussion. In fact not only the discussion whole manuscript should be written in the scientific principles (Clarity, brevity, chronological flow and attractiveness).

**Authors’ response**: We really do appreciate your comments and all what you have mentioned is right and accepted. We have modified the discussion and the whole manuscript.
